# Heterologous cross-seeding mimics cross-species prion conversion in a yeast model

**DOI:** 10.1186/1741-7007-7-26

**Published:** 2009-05-26

**Authors:** Namitha Vishveshwara, Susan W Liebman

**Affiliations:** 1 Department of Biological Sciences, Laboratory for Molecular Biology, Chicago, IL 60607, USA

## Abstract

**Background:**

Prions are self-perpetuating, infectious, aggregated proteins that are associated with several neurodegenerative diseases in mammals and heritable traits in yeast. Sup35p, the protein determinant of the yeast prion [*PSI*^+^], has a conserved C terminal domain that performs the Sup35p function and a prion domain that is highly divergent. Prions formed by chimeras of the prion domain of various species fused to the C domain of *Saccharomyces cerevisiae *exhibit a 'species barrier', a phenomenon first observed in mammals, and often fail to transmit the prion state to chimeras with prion domains of other species.

**Results:**

We focus on the chimera containing the prion domain of *Pichia methanolica *and examine how tight the 'species barrier' is between the chimera and *S. cerevisiae*. Although either of two Q/N-rich prions, [*PSI*^+^] or [*PIN*^+^], enhances the formation of the chimeric prion, [*CHI*^+^_PM_], neither a non-Q/N-rich prion nor a non-prion Q-rich aggregate promotes the formation of [*CHI*^+^_PM_]. [*CHI*^+^_PM_] has many features characteristic of yeast prions: aggregation, cytoplasmic transmission and a two-level protein structure. [*CHI*^+^_PM_] formed in the presence of [*PSI*^+^] can propagate independently of [*PSI*^+^] and forms at least two different variants of the prion, suggesting the generation and not transmission of new prion seeds.

**Conclusion:**

Although the sequence similarity between the *S. cerevisiae *Q/N-rich prion determinants and the *P. methanolica *prion domain is low, we find that the chimera containing the prion domain of *P. methanolica *can occasionally be cross-seeded by [*PSI*^+^] to mimic crossing the species barrier, to form the [*CHI*^+^_PM_] prion. Our data suggests that crossing the barrier occurs by a *de novo *formation of the foreign chimeric prion. Thus, the species barrier appears to be crossed by a heterologous seeding mechanism, wherein the infected prion protein uses the pre-existing seed as an inefficient template.

## Background

The idea that only nucleic acid elements could transfer genetic information was challenged by the discovery of prions [1]. Prions are the causative agents of several neurodegenerative diseases such as Creutzfeldt-Jakob disease (CJD) in humans, scrapie in sheep and bovine spongiform encephalopathy in cattle [2]. Once the prion protein (PrP) converts to its prion PrP^Sc ^form, which is in a largely β-sheet-rich, aggregated, amyloid state, it induces the conversion of normally folded, soluble, mainly α-helical PrP^C ^into the PrP^Sc ^form [3]. Thus, the protein structure is passed at the protein, and not at the nucleic acid level.

Although PrP^Sc ^converts PrP^C ^into the prion form, the propagation of the PrP^Sc ^amyloid is highly specific. PrP^Sc ^from one species can rarely convert PrP^C ^from another species into the prion form. This phenomenon, known as the species barrier, has been used to explain the lack of transfer of the disease from scrapie-infected sheep to humans [4]. However, a novel form of CJD is believed to have emerged from the conversion of human PrP to its prion form by ingested bovine prions [5]. Studies of laboratory PrP^Sc ^have shown that the some species barriers can be crossed whereas others are more rigid [4]. The barrier has been attributed not just to the difference in the primary sequence of PrP between species, but more importantly to the protein conformations that primary sequence is capable of adopting [6,7]. This difference in conformation of PrP^Sc ^is believed to give rise to different phenotypes of prion diseases, known as strains, that vary in characteristics such as time of incubation and patterns of neuropathology [8,9]. An *in vitro *study of mammalian proteins suggests that the barrier is crossed when the PrP primary sequence of a certain species is capable of adopting the conformation of a PrP^Sc ^strain of another species [7].

The phenomenon of information transfer through a protein-only process is not limited to mammals. Indeed, several prions have been studied in yeast [10]. Unlike mammals, where only one prion protein of unknown function has been described, yeast contain several prion protein determinants [11,12]. They share no homology with the primary sequence of PrP, but share common features such as high β-sheet content, infectivity and amyloid characteristics [13]. In addition, yeast prions also exist as different strains, called variants, that appear to differ in their amyloid conformations, which leads to phenotypic and biochemical variations [14-22].

Two extensively studied yeast prions [*PSI*^+^] and [*PIN*^+^] are altered, aggregated forms of Sup35p and Rnq1p, respectively [11,23,24]. Sup35p is a translation termination factor and the function of Rnq1p is unknown [23,25]. [*PSI*^+^] cells have reduced translation termination efficiency, as Sup35p is mainly in the aggregated form, whereas [*psi*^-^] cells that have non-prion Sup35p terminate efficiently [26]. Mendelian mutants of *SUP35 *also have reduced efficiency of translation termination, not because of aggregated Sup35p, but due to the mutation [27]. However, [*PSI*^+^] can be distinguished from *sup35 *mutants, as Sup35p is aggregated only in [*PSI*^+^] strains [28,29]. Sup35p has three distinct domains: a C-terminal domain (C) that performs the function of translation termination, and a N-terminal (N) and middle (M) domain that are required for the induction and faithful propagation of [*PSI*^+^] [14,29-32].

The [*PIN*^+^] prion is not a loss-of-function prion, but rather [*PIN*^+^] cells have the ability to induce [*PSI*^+^] more efficiently than [*pin*^-^] cells [33]. A cross-seeding model has been proposed to explain this phenomenon where the [*PIN*^+^] prion acts as an inefficient seed for the *de novo *formation of the [*PSI*^+^] prion [24,34]. Furthermore, different variants of [*PSI*^+^], analogous to mammalian PrP^Sc ^strains, are induced in [*PIN*^+^] cells by the overexpression of either the full length Sup35p or more efficiently by the Sup35NMp [14,29,35].

Like mammalian prions, yeast prions exhibit barriers across species [36-41]. [*PSI*^+^] formed by Sup35p from one species rarely passes the prion conformation to Sup35p from other species. Sup35p itself has retained its modular architecture in many species. Various species of yeast have the N, M and C domains where the sequence of the C domain is highly conserved between species, in contrast to the NM domains, which are often highly divergent [38]. However, the N domains of various species share the common features of having a high Q/N-rich content and oligopeptide repeats [38].

Chimeras of the NM from various species fused to the C domain of *Saccharomyces cerevisiae*, although able to form and propagate as prions in *S. cerevisiae*, fail to transmit the prion state to *S. cerevisiae *Sup35p or other chimeras [36,38,39]. This failure to pass the prion state is attributed to the highly divergent sequence of the NM domains. However, a certain variant of *S. cerevisiae *[*PSI*^+^] is able to transmit the prion state to the chimera of the NM of *Candida albicans *and C domain of *S. cerevisiae *(NM_CA_-C_SC_) [6] even though *S. cerevisiae *and *C. albicans *share only around 40% similarity in their NM domains. Thus, the variant of the prion is important for determining transmission across a species barrier. Other studies have shown that prion domains of other species, such as *S. bayanus *and *S. paradoxus*, which are much more similar to NM_SC_, and that co-aggregate with *S. cerevisiae *[*PSI*^+^] ([*PSI*^+^]_SC_), still exhibit low transmission of the prion state to these foreign species [39].

In this study we focus on the chimera of the NM of *Pichia methanolica *and C domain of *S. cerevisiae *(NM_PM_-C_SC_). NM_PM_-C_SC _as the sole copy of Sup35p can be induced and propagated in its prion state, [*CHI*^+^_PM_] (for chimeric [*PSI*^+^], PM for *P. methanolica*), in *S. cerevisiae *[36,37]. Like other chimeras it does not transmit its prion state *en masse *to *S. cerevisiae *Sup35p, although overexpression of NM_PM _can induce the formation of *S. cerevisiae *[*PSI*^+^], albeit at a lower frequency than by the overexpression of NM_SC_. However, overexpression of NM_SC _fails to induce NM_PM_-C_SC _into a prion. Prion conversion by overexpressed NM_PM _is specific to *S. cerevisia*e Sup35p as it fails to induce the prion form of chimeras from other species [38].

When NM_PM_-C_SC _or NM_CA_-C_SC _are expressed at the same level as *S. cerevisiae *Sup35p, neither the NM_PM_-C_SC _nor the NM_CA_-C_SC _chimeras are frequently infected by [*PSI*^+^]_SC _to become [*CHI*^+^_PM_] [38]. We examine how tight the barrier is and find that NM_PM_-C_SC _but not NM_CA_-C_SC _occasionally converts into [*CHI*^+^_PM_] in the presence of either of at least two Q/N-rich prions, [*PSI*^+^] or [*PIN*^+^]. We propose that the species barrier can be crossed by a heterologous seeding mechanism similar to that of the cross-seeding between the [*PIN*^+^] prion and Sup35p.

## Results

### NM_PM_-C_SC _but not NM_CA_-C_SC _is inactivated in the presence of [PSI^+^] at a frequency of 10^-4 ^to 10^-3^

**Figure 1:**
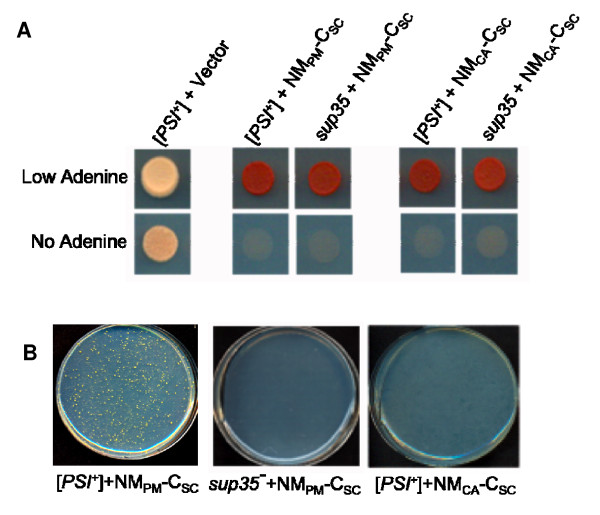
**NM_PM_-C_SC _but not NM_CA_-C_SC _is inactivated in the presence of [*PSI*^+^]**. **A**. NM_PM_-C_SC _and NM_CA_-C_SC _provide translation termination function. [*PSI*^+^] or *sup35 *cells ectopically expressing prion domains of either *Pichia methanolica *(NM_PM_) or *Candida albicans *(NM_CA_) fused to the C domain of *Saccharomyces cerevisiae *(NM_SC_) expressed on a *URA3 *plasmid on media containing low adenine-Ura and -Ade-Ura. -Ura is used to maintain the plasmid. **B**. NM_PM_-C_SC _is inactivated in [*PSI*^+^] cells and not in a *sup35 *mutant. Around 10^7 ^[*PSI*^+^] cells expressing either NM_PM_-C_SC _or NM_CA_-C_SC_, or *sup35 *mutant yeast expressing NM_PM_-C_SC _were plated on -Ade-Ura media.

As previously observed, chimeras of the prion domains of either *P. methanolica *(NM_PM_) or *C. albicans *(NM_CA_) fused to the C domain of *S. cerevisiae *(C_SC_) were functional in the presence of [*PSI*^+^] aggregates [38] (Figure 1A). This is phenotypically monitored using a yeast strain that has the *ade1-14 *allele with a suppressible nonsense mutation [26,42]. When the foreign fusions (containing an *HA *tag between *NM *and *C*) were ectopically expressed from a plasmid in [*PSI*^+^][*pin*^-^] *ade1-14 *cells at a moderate, constitutive level, the fusions remained functional and these cells were red on low adenine media (see Methods) and could not grow on -Ade (Figure 1A). To determine whether the fusions get inactivated occasionally, [*PSI*^+^] cells containing the fusions were plated onto plasmid selective -Ade media. The formation of Ade^+ ^colonies showed that the NM_PM_-C_SC _fusion was inactivated in [*PSI*^+^] cells at a frequency of about 10^-4 ^to 10^-3 ^(Figure 1B, Table 1). In contrast, the NM_CA_-C_SC _fusion was not inactivated in [*PSI*^+^] cells (Figure 1B). As a control these fusions were expressed in the presence of a *sup35 *mutant strain that, like [*PSI*^+^], can grow on -Ade, but unlike [*PSI*^+^] does not cause Sup35p to aggregate. The NM_PM_-C_SC _fusion was not inactivated in the *sup35 *mutant yeast (Figure 1A and 1B).

**Table 1:** Frequency of NM_PM_-C_SC _Ade^+ ^colonies in different yeast strains

Yeast Strain	Average frequency of Ade^+ ^colonies	95% confidence limit
Strong [*PSI*^+^]	6.1 × 10^-4^	(3–9.2) × 10^-4^

*sup35 *+ medium [*PIN*^+^]	1.5 × 10^-4^	(1–2) × 10^-4^

*sup35 *+ low [*PIN*^+^]	6.9 × 10^-5^	(5.2–8.7) × 10^-5^

*sup35 *+ HtQ103	<10^-7^	N/A

*sup35 *+ [Het-s]_y_	<10^-7^	N/A

*sup35*	<10^-7^	N/A

Frequency of Ade^+ ^colonies in different yeast strains was determined as described in Methods. N/A: Not applicable. Average and confidence limits were calculated using six independent transformants.

### NM_PM_-C_SC _Ade^+ ^colonies have prion properties

To determine if the inactivation of NM_PM_-C_SC _was due to its prionization, we tested for features characteristic of prions: aggregation and cytoplasmic inheritance. We denote NM_PM_-C_SC _in its active form as [*chi*^-^_PM_] and in its inactive form as [*CHI*^+^_PM_].

**Figure 2:**
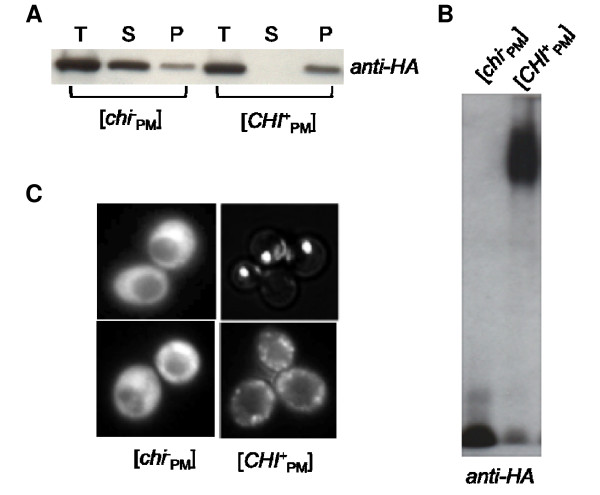
**Ade^+ ^colonies have prion properties**. **A**. NM_PM_-C_SC _is mainly in the pellet fraction in Ade^+ ^colonies. [*CHI*^+^_PM_] and [*chi*^-^_PM_] lysates subjected to high-speed centrifugation and probed for the chimeric protein using an *anti-HA *tag antibody. T: total; S: supernatant; P: pellet. **B**. [*CHI*^+^_PM_] colonies have SDS-stable sub-particles. [*CHI*^+^_PM_] and [*chi*^-^_PM_] lysates analyzed on an agarose gel and probed for the chimeric protein (*anti-HA *tag). **C**. NM_PM_-GFP forms puncta in [*CHI*^+^_PM_] colonies. NM_PM_-GFP under a copper-inducible promoter overexpressed in [*CHI*^+^_PM_] or [*chi*^-^_PM_] cells for 4 hours and examined under a fluorescence microscope. The two kinds of NM_PM_-GFP puncta are sometimes observable in the same [*CHI*^+^_PM_] culture and are not representative of different [*CHI*^+^_PM_] colonies.

If NM_PM_-C_SC _were in its prion form, most of the protein would be expected to be aggregated. The aggregation state was tested both biochemically and visually. When [*PSI*^+^] or [*PIN*^+^] cells are subjected to high-speed centrifugation most of the protein is in the pellet fraction, whereas in cells lacking the prion most of the protein is in the supernatant fraction [17,28,29]. Similarly, NM_PM_-C_SC _in [*CHI*^+^_PM_] lysates subjected to high-speed centrifugation was present in the pellet fraction and absent from the supernatant fraction (Figure 2A). In contrast, lysates from [*chi*^-^_PM_] cells had most of the NM_PM_-C_SC _in the supernatant fraction (Figure 2A).

The [*PSI*^+^] and [*PIN*^+^] prions are not dissolved into monomers when treated with sodium dodecyl sulfate (SDS) in the absence of boiling, but break into SDS-stable sub-particles that can be resolved on agarose gels [43,44]. Likewise, NM_PM_-C_SC _in [*CHI*^+^_PM_] lysates was not dissolved into monomers when treated with unheated 2% SDS, whereas NM_PM_-C_SC _from [*chi*^-^_PM_] lysates remain as monomers (Figure 2B).

*In vivo*, transiently overexpressed green fluorescent protein (GFP)-tagged prion domains are observed as distinct puncta in prion-containing cells, whereas cells lacking the prion show diffuse fluorescence [23,29,45]. Similarly, when NM_PM_-GFP was overexpressed transiently [*CHI*^+^_PM_] cells showed punctuate dots, whereas the fluorescence in [*chi*^-^_PM_] cells was diffuse (Figure 2C). Thus the chimeric protein (NM_PM_-C_SC_) is in an aggregated state in the Ade^+ ^colonies.

One of the characteristic features of prions is that they are passed from cell to cell through cytoplasmic mixing without a nuclear contribution. This is achieved by cytoduction, which involves mating donor and recipient yeast in the presence of a *kar1 *mutation that inhibits efficient nuclear fusion. Daughter cells with the recipient haploid nucleus and a mixture of the parental cytoplasms can be selected (cytoductants) [46]. When [*CHI*^+^_PM_][*PSI*^+^] *ade1-14 *(Ade^+^) cells were used as donors to cytoduce into a [*chi*^-^_PM_][*psi*^-^] *ade1-14 *recipient (Ade^-^) which, like the donors, was ectopically expressing NM_PM_-C_SC_, around 23% of the cytoductants displayed an Ade^+ ^phenotype (Table 2), whereas using [*chi*^-^_PM_][*PSI*^+^] as donor cells produced no Ade+ cytoductants (Table 2). Thus, the [*CHI*^+^_PM_] phenotype is transferred via cytoplasm. Taken together, it is clear that NM_PM_-C_SC _in the Ade^+ ^colonies obtained in the presence of [*PSI*^+^] was prionized.

**Table 2:** Frequency of Ade^+ ^cytoductants

**RECIPIENTS**	**DONORS**	
	[*CHI*^+^_PM_][*PSI*^+^] *ade1-14 *(Ade^+^)	[*chi*^-^_PM_][*PSI*^+^] *ade1-14 *(Ade^-^)

[*chi*^-^_PM_][*psi*^-^] *ade1-14 *(Ade^-^)	23.6 ± 16	0

[*chi*^-^_PM_]*Δsup35 ade1-14 *(Ade^-^)	12 ± 4.6	0

Cytoduction was performed as described in Methods. Five [*CHI*^+^_PM_][*PSI*^+^] donors and three [*chi*^-^_PM_][*PSI*^+^] donors were randomly chosen and approximately 50 cytoductants for each mating reaction were tested for the Ade^+ ^phenotype. Donors were isolated independently for each recipient. Standard deviation is shown.

### [CHI^+^_PM_] can propagate independently of [PSI^+^]

**Figure 3:**
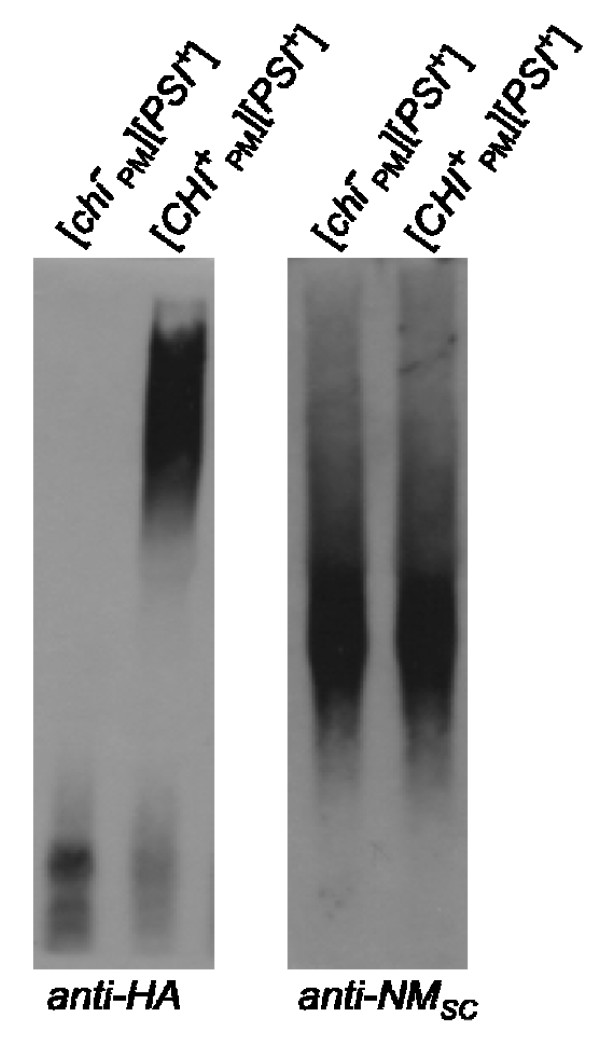
**NM_PM_-C_SC _is not incorporated into [*PSI*^+^] sub-particles**. [*chi*^-^_PM_][*PSI*^+^] and [*CHI*^+^_PM_][*PSI*^+^] lysates resolved on an agarose gel and probed for the chimeric protein (*anti-HA*) and reprobed for endogenous Sup35p (*anti-NM*_SC_).

Since [*CHI*^+^_PM_] aggregates exist in the presence of [*PSI*^+^] aggregates, we first asked whether NM_PM_-C_SC _could be incorporated into [*PSI*^+^] sub-particles. [*CHI*^+^_PM_][*PSI*^+^] and [*chi*^-^_PM_][*PSI*^+^] lysates were analyzed on an agarose gel and probed for the chimeric protein and re-probed for [*PSI*^+^] aggregates (Figure 3). The size distribution of [*PSI*^+^] and [*CHI*^+^_PM_] particles was distinct and the distribution of the [*PSI*^+^] particles was unchanged in the presence or absence of the [*CHI*^+^_PM_] sub-particles. This suggests that NM_PM_-C_SC _is not incorporated into [*PSI*^+^] sub-particles and forms independent sub-particles in the same cell. However, it does not rule out the possibility that a small number of NM_PM_-C_SC _protein molecules are incorporated in the [*PSI*^+^] sub-particles.

To check if [*CHI*^+^_PM_] propagation was dependent on the presence of [*PSI*^+^], cytoplasm from [*CHI*^+^_PM_][*PSI*^+^] and [*chi*^-^_PM_][*PSI*^+^] yeast was transferred (via cytoduction) into a strain lacking native *S. cerevisiae *Sup35p and being kept alive by a plasmid expressing NM_PM_-C_SC_. This strain cannot maintain [*PSI*^+^], as there is no source of *S. cerevisiae *Sup35p. When [*CHI*^+^_PM_][*PSI*^+^] yeast was used as the donor, around 12% of cytoductants were capable of growing on -Ade where as no Ade^+ ^cytoductants were obtained when [*chi*^-^_PM_][*PSI*^+^] donors were used (Table 2). All Ade^+ ^cytoductants that were tested had SDS-stable sub-particles and were cured by low amounts of guanidine hydrochloride (data not shown). Thus, [*CHI*^+^_PM_] formed in the presence of [*PSI*^+^] can propagate in the absence of [*PSI*^+^].

### [CHI^+ ^_PM_] forms variants in the presence of [PSI^+^]

**Figure 4:**
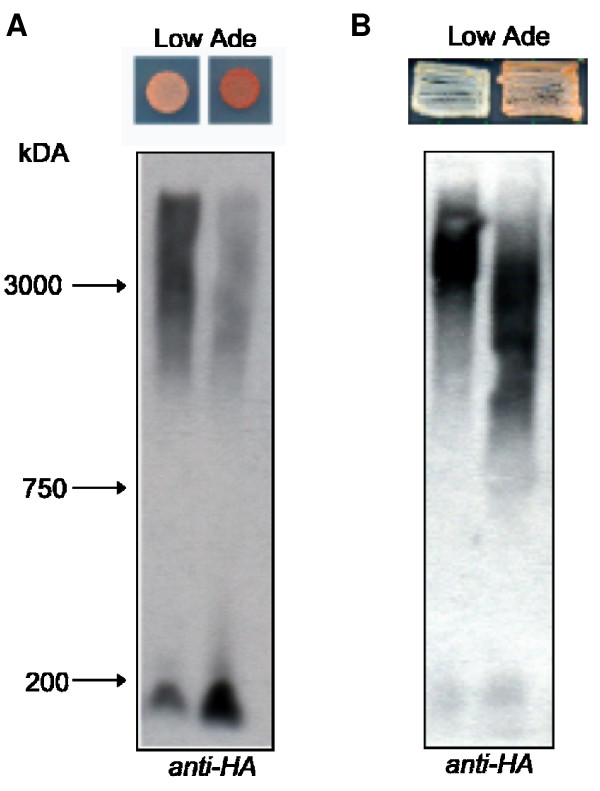
**More than one variant of [*CHI*^+^_PM_] forms in the presence of [*PSI*^+^]**. **A**. Some [*CHI*^+^_PM_] variants have differing amounts of SDS-stable sub-particles. Lysates from [*CHI*^+^_PM_] colonies differing in color on low adenine media were resolved on an agarose gel and probed with an *anti-HA *tag antibody that detects NM_PM_-C_SC_. Stained chicken pectoralis muscle extract provided molecular weight markers. The difference in amount of SDS-stable sub-particles was reproducible for this and another pair (not shown) isolated on the basis of color difference. **B**. Some [*CHI*^+^_PM_] variants have different sized SDS-stable sub-particles. Colonies formed on -Ade-Ura could be distinguished phenotypically on low adenine media by differences in color. Lysates from these variants were resolved on an agarose gel and probed with *anti-HA *tag antibody, which detects NM_PM_-C_SC_. The difference in size of SDS-stable sub-particles was reproducible for this and another pair (not shown) isolated on the basis of color difference.

When Sup35p from *S. cerevisiae *is ectopically expressed in a [*PSI*^+^] strain, it efficiently joins the pre-existing [*PSI*^+^] aggregate and maintains that particular variant [38]. However, when [*PSI*^+^] is formed *de novo *in the presence of the heterologous prion [*PIN*^+^], it forms many different variants that can be distinguished phenotypically [14]. Strong [*PSI*^+^] variants suppress *ade1-14 *mutation better than weak [*PSI*^+^] variants, causing the former to be white and the latter pink on complete media. In the presence of [*PSI*^+^] at least two variants of [*CHI*^+^_PM_] could be distinguished phenotypically, by the different colors on media containing low amounts of adenine (Figure 4). These variants, as in the case of [*PSI*^+^], could sometimes be differentiated biochemically by the size of their sub-particles [43] (Figure 4B). Unlike [*PSI*^+^] variants but like at least one strong hybrid [*CHI+*_PM_] variant (where NM_PM_-C_SC _is the sole copy of Sup35p in the cell), the strong chimeric variant was associated with the larger sub-particles whereas the small sub-particles were associated with the weaker variant (Figure 4B) [43]. Some strong and weak [*CHI*^+^_PM_] variants distinguished on the basis of color could not be differentiated by a change in the size of sub-particles, but did differ in the amount of sub-particles present (Figure 4A).

### [CHI^+ ^_PM_] formation is enhanced by the [PIN^+^] prion but not by poly Q aggregates or the non-QN-rich prion [Het-s]_y_

**Figure 5:**
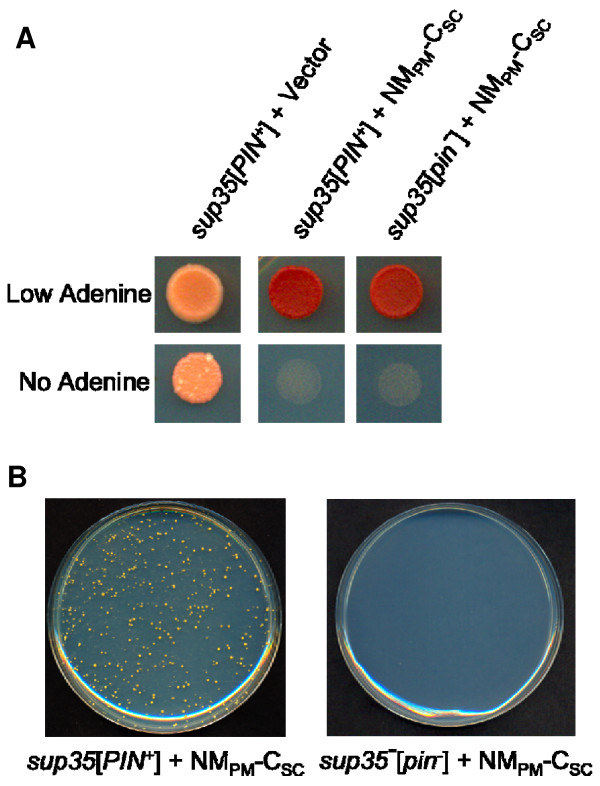
**[*PIN*^+^] enhances [*CHI*^+^_PM_] formation**. **A**. NM_PM_-C_SC _provides translation termination function to a *sup35 *mutant containing the medium [*PIN*^+^] variant. *sup35 *medium [*PIN*^+^] or [*pin*^-^] yeast ectopically expressing NM_PM_-C_SC _on media containing low or no adenine without uracil (to maintain the plasmid). Cells with the medium [*PIN*^+^] variant are shown here. NM_PM_-C_SC _is also functional in *sup35 *mutant yeast with the low [*PIN*^+^] variant. **B**. NM_PM_-C_SC _is inactivated in *sup35 *mutant cells containing the medium or low [*PIN*^+^] variant and not in a [*pin*^-^] *sup35 *mutant Around 10^7 ^*sup35 *mutant cells containing either medium [*PIN*^+^] or [*pin*^-^] expressing NM_PM_-C_SC _were plated onto -Ade-Ura. The frequency of Ade^+ ^colonies was estimated in *sup35 *yeast with medium and low [*PIN*^+^] variants. The Ade^+ ^frequency in *sup35 *yeast with high and very high [*PIN*^+^] variants was not determined as suppression in these strains is enhanced compared with *sup35 *[*pin*^-^] yeast.

As the formation of [*CHI*^+^_PM_] resembled the *de novo *formation of [*PSI*^+^] in the presence of [*PIN*^+^], we asked if [*PIN*^+^] could enhance the formation of [*CHI*^+^_PM_]. Variants of [*PIN*^+^] were cytoduced into a *sup35 *mutant, as the change from the [*chi*^-^_PM_] to the [*CHI*^+^_PM_] state cannot be monitored in wild type [*PIN*^+^][*psi*^-^] yeast as native Sup35p is functional in these strains. Similar to its expression in [*PSI*^+^] strains, the chimeric protein was functional in the presence of all the [*PIN*^+^] variants (Figure 5A and data not shown). To determine if NM_PM_-C_SC _was inactivated occasionally, *sup35 *mutants with [*PIN*^+^] variants (or [*pin*^-^]) expressing NM_PM_-C_SC _were plated on plasmid selective -Ade media. NM_PM_-C_SC _in *sup35 *mutants containing [*PIN*^+^] variants was inactivated at a frequency of approximately 10^-5 ^to 10^-4^, whereas NM_PM_-C_SC _in *sup35 *[*pin*^-^] yeast remained functional (Figure 5B, Table 1). Ade^+ ^colonies contained SDS-stable aggregates of NM_PM_-C_SC_, indicating that the chimeric protein converted to its prion form in the presence of [*PIN*^+^] (data not shown).

As two Q/N-rich prions, [*PSI*^+^] and [*PIN*^+^], enhanced the formation of [*CHI*^+^_PM_], we tested whether an aggregated, non-prion protein could enhance the formation of [*CHI*^+^_PM_]. The N-terminal region of the Huntingtin protein with expanded polyglutamine repeats (either 72 or 103 repeats: HtQ72, HtQ103), which is associated with Huntington's disease, fused to GFP was expressed in a *sup35 *mutant strain along with NM_PM_-C_SC_. HtQ72-GFP and HtQ103-GFP, shown to aggregate in wild type strains [47], also aggregated in the *sup35 *mutant, but failed to enhance the conversion of NM_PM_-C_SC _from a [*chi*^-^_PM_] to a [*CHI*^+^_PM_] state (Table 1). We also tested whether a non-Q/N-rich prion, [Het-s]_y_, a prion from *Podospora anserine *that can propagate as a prion in yeast [48], could enhance the formation of [*CHI*^+^_PM_]. The prion domain of HET-s fused to GFP (the protein determinant of [Het-s]_y_), was induced into the prion form ([48] and see Methods) in a *sup35 *mutant expressing NM_PM_-C_SC_, but failed to enhance the formation of [*CHI*^+^_PM_] (Table 1).

## Discussion

Although the degree of similarity is not high between the prion domains of *S. cerevisiae *and either *P. methanolica *(approximately 32%) or *C. albicans *(approximately 40%), we show here that *in vivo *the chimeric protein NM_PM_-C_SC_, but not NM_CA_-C_SC _occasionally prionizes in the presence of [*PSI*^+^] (10^-4 ^to 10^-3^). We show that NM_PM_-C_SC _does not spontaneously prionize in the absence of the [*PSI*^+^] seed, as it does not form a prion in a *sup35 *mutant. We determine that NM_PM_-C_SC _does indeed prionize (called [*CHI*^+^_PM_]) in [*PSI*^+^] cells by showing that [*CHI*^+^_PM_] cells possess characteristic features of yeast prions. NM_PM_-C_SC _in [*CHI*^+^_PM_] cells is aggregated as NM_PM_-GFP formed puncta in [*CHI*^+^_PM_] and not [*chi*^-^_PM_] cells. Furthermore, biochemically [*CHI*^+^_PM_] also shares the two-level structural organization of the [*PIN*^+^] and [*PSI*^+^] yeast prions [43,44]: NM_PM_-C_SC _appears to be assembled into large aggregates (resolved by high-speed centrifugation) that can be broken into SDS-stable sub-particles. Additionally, we show the [*CHI*^+^_PM_] phenotype can be transferred via cytoplasm, which is common to all known yeast prions [11,23,24,42,49]. Thus, we show that although the transmission of the prion state across species barriers is not very efficient, the prion state can be transferred occasionally to give rise to a foreign prion.

When two prions exist in the same cell, it is possible for them to form tight interactions or propagate as separate entities. Since [*CHI*^+^_PM_] was formed in the presence of [*PSI*^+^] we asked whether NM_PM_-C_SC _molecules are incorporated into [*PSI*^+^] sub-particles. If this were the case, then the mixed sub-particles might have a size distribution distinct from that of [*PSI*^+^] in the absence of [*CHI*^+^_PM_]. As the size distribution of the [*PSI*^+^] sub-particles is unchanged, and [*CHI*^+^_PM_] sub-particles are a different size distribution, this suggests that NM_PM_-C_SC _is not incorporated into [*PSI*^+^] sub-particles. However, this shift would be observable only if the number of NM_PM_-C_SC _molecules incorporated into [*PSI*^+^] sub-particles is fairly large, thus not excluding the possibility that a small number of NM_PM_-C_SC _molecules might be incorporated. This independence of sub-particles is similar to [*PIN*^+^] and [*PSI*^+^] that were shown to form independent sub-particles in the same cell [44]. While the *de novo *formation of [*PSI*^+^], by the overexpression of Sup35 or Sup35NM, is dependent on the presence of [*PIN*^+^], the propagation of [*PSI*^+^] is independent of [*PIN*^+^] [33,50]. Similarly, the formation of [*CHI*^+^_PM_] is dependent on [*PSI*^+^], but [*PSI*^+^] does not incorporate NM_PM_-C_SC _into its sub-particles and [*CHI*^+^_PM_] can indeed propagate independently of [*PSI*^+^]. However, unlike *de novo *formation of [*PSI*^+^], [*CHI*^+^_PM_] is formed in the absence of overexpression of NM_PM_-C_SC_.

During the *de novo *formation of [*PSI*^+^] by the overexpression of Sup35p or Sup35NMp in [*PIN*^+^] cells, several variants of [*PSI*^+^], distinguished by phenotypic differences, are formed [14]. Analogously, variants of [*CHI*^+^_PM_] differing in phenotype and biochemically are formed in the presence of [*PSI*^+^], although NM_PM_-C_SC _is not overexpressed. We suggest that [*PSI*^+^] cross-seeds [*CHI*^+^_PM_], similar to [*PIN*^+^] cross-seeding the formation of [*PSI*^+^]. Since [*PSI*^+^] forms separate sub-particles from those of [*CHI*^+^_PM_], this suggests that *S. cerevisiae *Sup35p and NM_PM_-C_SC _do not form tight interactions within the cell. Furthermore, overexpressed NM_PM_-GFP and Sup35NM_SC_-GFP do not co-aggregate in the cell [39], suggesting that interaction between endogenous Sup35p and the chimeric protein is limited. Species that have NM domains that are much more similar to *S. cerevisiae*, such as *S. bayanus *and *S. paradoxus*, have been shown to co-aggregate but there is no transmission of the prion state [39]. This suggests that tight interactions might actually hamper the formation of the heterologous prion and that heterologous prion formation might be mediated by transient interactions between the seed and the prionizing protein. Indeed, *in vitro *PrP in the non-fiber form from one species is capable of binding PrP in the fiber form of another species, but there is no conversion of the non-fiber form to the fiber form [51]. Thus, stable interactions might actually be inhibitory to the process of heterologous prion conversion.

As [*CHI*^+^_PM_] seems to be cross-seeded by [*PSI*^+^] we determined whether [*CHI*^+^_PM_] could be seeded by [*PIN*^+^], another Q/N-rich prion. Surprisingly, the prion domain of Rnq1p, that has even less similarity than *S. cerevisiae *Sup35p to NM_PM_(15%), is able to seed NM_PM_-C_SC _to give rise to [*CHI*^+^_PM_], albeit at a lower frequency than in the presence of [*PSI*^+^] (approximately 10^-5 ^to 10^-4^). Importantly, unlike the [*PIN*^+^]-promoted induction of [*PSI*^+^] which requires overexpression of Sup35p or at least its prion domain, the appearance of [*CHI*^+^_PM_] in the presence of either [*PIN*^+^] or [*PSI*^+^] occurs in the absence of NM_PM_-C_SC _overexpression. Interestingly, the property of enhancing [*CHI*^+^_PM_] formation seems limited to the two Q/N-rich prions that we tested. Aggregates of GFP fused to HtQ103p, the mutated first exon of the Huntingtin protein (HtQ103), whose aggregation is associated with Huntington's disease [52] and enhances *de novo *formation of [*PSI*^+^] [34], fail to enhance the formation of [*CHI*^+^_PM_]. Furthermore, a non-Q/N-rich prion [Het-s]_y_, a prion from *P. anserine *that can propagate in yeast, that is induced twofold higher in [*PIN*^+^] cells [48], also failed to enhance [*CHI*^+^_PM_] formation, suggesting that although Q/N-rich prions can enhance the formation of non-Q/N-rich prions, non-Q/N-rich prions do not always enhance the formation of Q/N-rich prions *in vivo*. In fact, *in vivo *non-Q/N-rich amyloids failed to enhance the *de novo *formation of [*PSI*^+^] [34], suggesting that cross talk between Q/N-rich and non-Q/N-rich prions might occur only in one direction.

Two models have been proposed to explain the ability of heterologous prions to enhance the *de novo *formation of other prions: titration of inhibitory factors by the heterologous prion, and direct cross-seeding by the heterologous prion [24,34,53]. One or both of these mechanisms could be playing a role in the cross-seeding activity. *In vitr*o evidence supports the direct cross-seeding model, as many Q/N-rich and non-Q/N-rich amyloids have been shown to stimulate the aggregation of Sup35p [34]. Since [*CHI*^+^_PM_] formation is specific to Q/N-rich prions, we suggest that *de novo *formation of this prion requires interactions between Q/N-rich domains. Several studies have shown that Q and N residues play an important role in the initial step of amyloid formation [54], which might be essential for the formation of [*CHI*^+^_PM_]. Since we see no increase in [*CHI*^+^_PM_] formation in the presence of the non-prion Q/N-rich HtQ103 amyloid, we propose that interaction of resident prion propagating/enhancing factors that associate with the prion cross-seed might help stabilize the newly forming [*CHI*^+^_PM_] seed. For example Hsp104, a chaperone required for the propagation of all known yeast prions [12,26,33,55], binds preferentially to [*PSI*^+^] aggregates *versus *non-prion Sup35p [56]. As [*CHI*^+^_PM_] seeds are probably first formed by an interaction with the [*PSI*^+^] seed, Hsp104 bound to [*PSI*^+^] might play a role in propagating newly formed [*CHI*^+^_PM_] seeds.

Although prion variants play an important role in the transmission of the prion state across a species barrier, two studies show that variants cause slightly differing results. In the case of mammalian *in vitro*-made fibers, Syrian hamster PrP (23–144) fibers were able to cross-seed mouse PrP (23–144) protein but not vice versa. However, Syrian hamster-seeded mouse fibers had properties of the Syrian hamster seed and not that of spontaneously formed mouse fiber [7]. In the case of yeast, one specific variant of [*PSI*^+^]_SC _was able to cross the barrier and infect the NM domain of *C. albicans *(NM_CA_) to give rise to a novel variant of the prion form of NM_CA_-C_SC _[6]. We see that although NM_PM _and NM_SC _have very little similarity, NM_PM_-C_SC _is infected by [*PSI*^+^]_SC _to give rise to not one but at least two variants of [*CHI*^+^_PM_]. We suggest that the chimeric foreign protein NM_PM_-C_SC _is heterologously cross-seeded to form [*CHI*^+^_PM_] *de novo*, giving rise to different variants. In the case of homologous seeding of Sup35p to form [*PSI*^+^], studies have shown that short peptide sequences mediate initial nucleation to give rise to amyloid fibers [57]. We propose that the variant of the infecting prion is important as different peptide sequences may be exposed that allow different foreign protein sequences to interact to lead to prionization. Thus the [*PIN*^+^] prion, that has such low similarity with NM_PM_, might have short stretches of peptides that can interact with NM_PM _giving rise to [*CHI*^+^_PM_]. Our data suggest that heterologous seeding events between proteins from different species might mimic a crossing of the species barrier.

## Conclusion

We show here that in spite of low sequence similarity between the *P. methanolica *prion domain and the *S. cerevisiae *Q/N-rich prion determinants, the chimera can convert to its prion form, [*CHI*^+^_PM_], in the presence of [*PSI*^+^] and [*PIN*^+^]. [*CHI*^+^_PM_] has many characteristics of yeast prions such as aggregation and transmission by cytoplasmic mixing. Interestingly, more than one variant of [*CHI*^+^_PM_] was isolated in the presence of [*PSI*^+^]. These results suggest that [*CHI*^+^_PM_] is formed anew, similar to the *de novo *formation of [*PSI*^+^] in the presence of [*PIN*^+^] by the overexpression of Sup35p. Thus, heterologous seeding events leading to newly formed chimeric prion variants might mimic the crossing of the species barrier.

## Methods

### Plasmids

Centromeric plasmids pNM_PM_-C_SC _(p1180) and pNM_CA_-C_SC _(p1072) (kindly provided by Jonathan Weissman) have NM domains of the following species – PM: *P. methanolica*, CA: *C. albicans*, fused to the C domain of *S. cerevisiae *(C_SC_), with an HA tag between the NM and C domains, under the *SUP35 *promoter, in pRS316 (*URA3*) [58]. The control vector used was pRS316 [58].

pNM_PM_-GFP (p1680) is a centromeric (*URA3*) plasmid in which the copper-inducible promoter controls the expression of NM_PM_-GFP (kindly provided by Yury Chernoff). NM_PM_-GFP was induced using 50 μM copper for 4 hours and observed using a Zeiss AxioScope2.

*HtQ72-GFP *(p1292) and *HtQ103-GFP *(p1293) were expressed from the constitutive *GPD *promoter in the high copy pRS425 plasmid [58] (*LEU2*, 2 μ) (kindly provided by Susan Lindquist). The control vector for these constructs was pRS425 [58].

pHET-s(PrD)-GFP (p1393) contains the HET-s prion domain (PrD) fused to GFP under a galactose-inducible promoter in a centromeric *TRP1 *vector [48].

### Yeast strains and media

The following yeast strains are derivatives of 74-D694 (*MATa ade1-14 leu2-3,112 his3-Δ200 trp1-289 ura3-52*) [35]: L1763 contains strong [*PSI*^+^] and is [*pin*^-^] [33]; L2333 is a *sup35 *mutant containing two point mutations in the C terminus [59]; L2802 is a *can1*^*R*^, [*rho*^-^] version of L2333, where *can1*^*R *^is a recessive marker. L2598, used for cytoduction, is a GuHCl-cured, [*rho*^-^], *kar1-15 *version of L2176 (*MATα ade1-14 leu2-3,112 his3-Δ200 trp1-289 ura3-52*) [17]. L2802 was cytoduced with different variants of [*PIN*^+^] to give rise to: L2937, low [*PIN*^+^]; L2938, medium [*PIN*^+^]; L2939, very high [*PIN*^+^]; L2940, high [*PIN*^+^]; L2941, [*pin*^-^]. L2958 (*MATα ade1-14 leu2-3,112 his3-Δ200 trp1-289 ura3-52 sup35::TRP1 kar1 cyh*^*R*^) is a *sup35Δ *strain being kept alive by pNM_PM_-C_SC_.

Standard yeast media and cultivation procedures were used [60]. Transformants were grown on synthetic dextrose (SD) lacking the appropriate amino acid. To monitor the efficiency of translational read through of *ade1-14 *transformants containing *SUP35 *fusion proteins, the color of cells grown on plasmid-selective, low adenine media with 0.13% yeast nitrogen base, 0.5% ammonium sulfate, 1% casamino acids, and 2% glucose, tryptophan, one quarter the required amount of adenine and no uracil was determined. This same media but with additional adenine and 5 mM guanidine hydrochloride (GuHCl) was used to cure the [*CHI*^+^_PM_] prion. Synthetic glycerol (SG) -Ura containing 3 mg/liter cyclohexamide was used to select for cytoductants.

To isolate *can1*^*R *^mutants, cells were plated on SD-Arg containing 60 mg/liter canavanine and resistant colonies were picked. To make the strain [*rho*^-^], cells were grown on complex glucose media (YPD) containing 0.05 mg/ml ethidium bromide [61]. To select for *sup35 *mutant cytoductants with different [*PIN*^+^] variants, SG-Arg + canavanine was used.

### Scoring for the formation of [CHI^+ ^_PM_]

Previously described suppression assays were used to score for the formation of [*CHI*^+^_PM_] [26]. Briefly, in [*PSI*^+^] strains the premature stop codon in the *ade1-14 *allele is read through, allowing *ade1-14 *cells to grow on -Ade and causing them to be white on YPD. This is because most of the Sup35p is inactivated in [*PSI*^+^] cells because it is sequestered into the prion aggregate. In [*psi*^-^] cells, Sup35p is available for efficient translation termination and thus [*psi*^-^] *ade1-14 *cells do not grow on -Ade and are red on YPD. A *sup35 *mutant (L2333) is also able to read through the *ade1-14 *premature stop codon, due to a Mendelian mutation in the *SUP35 *gene that impairs the activity of the Sup35p protein, and thus can grow on -Ade. L1763 (strong [*PSI*^+^][*pin*^-^]), L2333 (*sup35 *mutant) or L2333 with [*PIN*^+^] variants were transformed with plasmids (pNM_PM_-C_SC_, pNM_CA_-C_SC_, and control vector) and transformants were dissolved in water and spotted onto SD-Ura (to maintain the plasmid), low adenine (to monitor read through via color) or SD-Ura-Ade media (to monitor read through with growth on -Ade). This determined the functionality of NM_PM_-C_SC _in the [*PSI*^+^] yeast. However, if a few NM_PM_-C_SC _molecules were inactivated, it would be difficult to monitor this in the above spot test. To test if NM_PM_-C_SC _was occasionally inactivated, cells taken from SD-Ura were dissolved in water and serially diluted. Larger numbers of cells (10^4 ^to 10^7^) were plated onto SD-Ade-Ura and lower dilutions (10^1 ^to 10^3^) were plated onto SD-Ura. Viability was determined by the number of cells on SD-Ura and the frequency of formation of [*CHI*^+^_PM_] was determined by comparing the number of colonies on SD-Ura-Ade to the number on SD-Ura.

### Effect of [Het-s]_y _on [CHI^+ ^_PM_] formation

[Het-s]_y _was essentially induced and maintained as previously reported [48]. Briefly, pHET-s(PrD)-GFP was transformed into a *sup35 *mutant and transformants were grown on synthetic raffinose (SR)-Trp + 2% galactose to maintain the plasmid and induce HET-s(PrD)-GFP. This was then transferred to SR-Ura-Trp + 0.05% galactose to maintain [Het-s]_y _as dots. Either dot or diffuse HET-s(PrD)-GFP cells were micromanipulated and propagated on SR-Ura-Trp + 0.05% galactose. NM_PM_-C_SC _or a control vector were transformed into [Het-s]_y_-containing strains and plated on SR-Ura-Trp + 0.05% galactose with or without adenine to monitor the formation of [*CHI*^+^_PM_]. Cells with Het-s(PrD)-GFP dots retained dots in 70 to 90% of the cells whereas diffuse cells remained diffuse.

### Cytoduction

Donor [*PSI*^+^][*CHI*^+^_PM_] or [*PSI*^+^][*chi*^-^_PM_] strains were mated to L2598, a *cyh*^*R *^strain defective in nuclear fusion (*kar1*). Both the donor and recipient strains contained pNM_PM_-C_SC_, expressing NM_PM_-C_SC_. Mating was done on SD-Ura to maintain pNM_PM_-C_SC_. Cytoductants were selected on SG-Ura + 3 mg/liter cyclohexamide. This media selects against the donor and diploids, as they cannot grow in the presence of cyclohexamide. Recipient cells cannot grow on SG as they lack mitochondria ([*rho*^-^]). Only cytoductants, recipient cells that have acquired mitochondria (and therefore cytoplasm) from the donor, can grow. To test whether [*CHI*^+^_PM_] can be maintained independently of [*PSI*^+^], [*PSI*^+^][*CHI*^+^_PM_] or [*PSI*^+^][*chi*^-^_PM_] donor strains were cytoduced into L2958. Cytoductants were selected on SG-Ura + 3 mg/liter cyclohexamide.

### Protein analysis

To prepare cell lysates, strains were grown in appropriate media and harvested at an optical density of 1.5 to 2.0 (A_600_). Crude cell lysates were prepared by physical disruption using glass beads (0.5 mm, Biospec, Bartlesville, OK, USA) in 750 μl of lysis buffer containing 50 mMTris/HCl, pH 7.5, 50 mM KCl, 10 mM MgCl_2 _and 5% (w/v) glycerol with protease inhibitor cocktail (P8215, 1:50, Sigma, St. Louis, MO, USA) and 5 mM PMSF. Cells were lysed by vortexing (Vortex-Genie 2) at high speed three times for 2 min each with 1 min in between in ice at 4°C. Crude lysates were pre-cleared by centrifuging at 3000 *g *for 5 min at 4°C to remove unlysed cells. The aqueous layer was used for further analysis.

To perform high-speed centrifugation analysis, approximately 600 to 800 μg of crude cell lysate in 300 μl was spun at 100,000 *g *for 30 min at 4°C. The supernatant was separated from the pellet fraction and the pellet fraction was dissolved in 300 μl of lysis buffer with protease inhibitor cocktail (Sigma, St. Louis, MO, USA) supplemented with 5 mM PMSF. Approximately 30 μl of the supernatant, the pellet and total protein each were mixed with 4× sample buffer (final concentration 62.5 mM Tris pH 6.8, 5% glycerol, 2% SDS and 0.2% bromophenol blue) and 2% β-mercaptoethanol and boiled for 5 min. This was then subjected to polyacrylamide gel electrophoresis using BioRad 10% Tris-HCl ready gels and transferred to a PVDF membrane. The NM_PM_-C_SC _was detected using a monoclonal mouse *anti-HA *tag antibody (1:10,000, Sigma Aldrich, St. Louis, MO, USA).

To perform semi-denaturing detergent agarose gel electrophoresis (SDD-Age) analysis, crude lysate (40 to 80 μg of total protein) was treated with 2% SDS in sample buffer for 7 min at room temperature. The lysates were subjected to agarose electrophoresis on a 1.5% agarose gel in running buffer to resolve the [*CHI*^+^_PM_] sub-particles, and were transferred to a PVDF membrane using a wider mini-gel cassette or a semi-dry blot. Native Sup35p was detected by rabbit *anti-NM*_SC _antibody (kindly provided by S. Lindquist). The PVDF membrane was stripped prior to probing for the native Sup35p using the Applied Biosystems protocol. A preparation of chicken pectoralis extract (a kind gift from T. Keller) was used to estimate molecular weight [62]. When stained with Coomassie, chicken pectoralis extract reveals several abundant muscular proteins: titin (3,000 kDa), nebulin (750 kDa), and myosin heavy chain (200 kDa). Although this ladder cannot be used for precise determination of molecular mass, it does provide an estimate.

## Authors' contributions

NV and SWL conceived and designed the experiments, analyzed data, and wrote the manuscript. NV performed the experiments and collected data. Both authors read and approved the final manuscript.
